# Creutzfeldt–Jakob Disease and Fatal Familial Insomnia: Demographics and In-Hospital Mortality in Spain

**DOI:** 10.3390/jcm13154401

**Published:** 2024-07-27

**Authors:** Natividad Cuadrado-Corrales, Ana Lopez-de-Andres, Valentín Hernández-Barrera, Javier De-Miguel-Díez, Ana Jimenez-Sierra, David Carabantes-Alarcon, Jose J. Zamorano-Leon, Rodrigo Jimenez-Garcia

**Affiliations:** 1 Department of Public Health & Maternal and Child Health, Faculty of Medicine, Universidad Complutense de Madrid, 28040 Madrid, Spain; mariancu@ucm.es (N.C.-C.); dcaraban@ucm.es (D.C.-A.); josejzam@ucm.es (J.J.Z.-L.); rodrijim@ucm.es (R.J.-G.); 2 Preventive Medicine and Public Health Teaching and Research Unit, Health Sciences Faculty, Rey Juan Carlos University, 28922 Alcorcón, Spain; valentin.hernandez@urjc.es; 3 Respiratory Care Department, Hospital General Universitario Gregorio Marañón, Instituto de Investigación Sanitaria Gregorio Marañón (IiSGM), Universidad Complutense de Madrid, 28007 Madrid, Spain; javier.miguel@salud.madrid.org; 4 Faculty of Medicine, Universidad San Pablo Ceu, 28668 Madrid, Spain; a.jimenez100@usp.ceu.es

**Keywords:** Creutzfeldt–Jakob disease, fatal familial insomnia: hospitalizations, epidemiology, mortality, costs

## Abstract

**Background:** Creutzfeldt–Jakob disease (CJD) and fatal familial insomnia (FFI) are prion diseases characterized by severe neurodegenerative conditions and a short duration of illness. **Methods**: This study explores the characteristics of hospitalizations for CJD and FFI in Spain from 2016 to 2022 using the Spanish National Hospital Discharge Database (SNHDD). **Results**: We identified a total of 1063 hospital discharges, including 1020 for CJD and 43 for FFI. Notably, the number of hospitalized patients with FFI showed a significant peak in 2017. The average length of hospital stay (LOHS) was 13 days for CJD and 6 days for FFI, with in-hospital mortality rates (IHM) of 36.37% for CJD and 32.56% for FFI. Among CJD patients, the average LOHS was 14 days, with a significantly longer duration for those who experienced IHM. **Conclusions**: The presence of sepsis or pneumonia and older age were associated with a higher IHM rate among CJD patients. The total estimated cost for managing CJD and FFI patients over the study period was EUR 6,346,868. This study offers new insights into the epidemiology and healthcare resource utilization of CJD and FFI patients, which may inform future research directions and public health strategies.

## 1. Introduction

Transmissible spongiform encephalopathies (TSE), also known as prion diseases, constitute a heterogeneous group of fatal neurological disorders characterized by the accumulation of abnormal isoforms of prion proteins in the brain [1]. The key molecular event in the pathogenesis of TSEs is the conversion of the cellular prion protein PrPC into a disease-associated pathological isoform, PrPSc [2]. This conversion supports the notion that PrPSc is the infectious agent, adopting various structures that represent different prion strains [3]. The presence of PrPSc in the brain induces a cascade of detrimental effects, including synaptic loss, neuronal death, the development of spongiform neuronal changes, and gliosis, all of which lead to neurodegeneration [4].

Despite their varied etiologies, all prion diseases accumulate infectious protein in the brain, which leads to severe neurological symptoms [5]. Creutzfeldt–Jakob disease (CJD) is the most frequent etiology, accounting for approximately 85% of cases [6]. In CJD, these pathological changes manifest as a fulminant, severe, and fatal progression of neurological symptoms, including rapidly progressive cognitive impairment, dementia, behavioral disturbances, impairment of higher cortical functions, and myoclonus. In the terminal stages, akinetic mutism is prevalent [7,8]. The World Health Organization (WHO) has established rigorous diagnostic criteria for Sporadic Creutzfeldt–Jakob Disease (sCJD), categorized into different levels of diagnostic certainty [9]. Among these, “probable” diagnostic certainty is achieved in patients with rapidly progressive dementia and the presence of specific biomarkers. Notably, these biomarkers include the detection of 14-3-3 protein in cerebrospinal fluid (CSF) [10] and the real-time quaking-induced conversion (RT-QuIC) assay [11]. The 14-3-3 protein is a marker indicative of neuronal damage [12], while the RT-QuIC assay is a technique that amplifies abnormal prion proteins, allowing for more precise and early detection of CJD [13]. The use of these assays in patients with suspected sCJD is crucial for improving diagnostic certainty.

Fatal familial insomnia (FFI) is a neurodegenerative prion disease characterized by substantial atrophy in the thalamic nucleus, which plays a key role in the development of clinical features and characteristics [14]. It is almost always genetically inherited, caused by mutations in the *PRNP* gene, which encodes the prion protein [15]. FFI affects notably younger age groups and presents a severe and rapidly progressive course, leading to a fatal outcome, characterized by progressively worsening insomnia, autonomic dysfunction, cognitive decline, and motor system disturbances [7,16].

The global incidence of prion disease is typically reported to be around 1–2 cases per million people per year, with significant variations between countries; this has increased considerably over the years. This trend could be explained by an improved surveillance system and progressive aging of the population [17]. Cases with genetic and accidentally transmitted etiologies, on the other hand, have either remained constant in number or decreased. In Spain, the overall incidence of CJD is slightly higher than 1.28 cases per million [18], constituting 89% of all prion diseases. Additionally, FFI accounts for 5% of all prion diseases [19]. The prevalence of FFI in Spain is higher compared to other countries, possibly due to a founder effect associated with the mutation in the northern region of Spain [20].

Patients diagnosed with prion diseases are affected by the comorbid conditions that are typical of older age, such as Alzheimer’s disease, Parkinson’s disease, tauopathies, frontotemporal dementia, and cerebrovascular disease [21]. Affected patients may also present with comorbidities such as diabetes, kidney disease, chronic pulmonary disease, malignancies, myocardial infarction, and mild liver diseases. The average length of hospital stay (LOHS) is slightly over eight days, with an in-hospital mortality rate exceeding 50% [22].

Assessing the circumstances of hospitalization with prion diseases could help to optimize the healthcare system [23], which is a fundamental part of improving public health strategies [24]. These diseases represent a significant public health problem due to their infectious nature. Unlike many common infectious diseases, prions are highly resistant to conventional sterilization and disinfection methods, complicating outbreak management and environmental protection in healthcare settings [25]. Additionally, prion diseases often have a long incubation period and rapid progression once symptoms appear, leading to delays in diagnosis and a high mortality rate once the disease manifests [26]. These features, combined with their low prevalence, contribute to a significant burden on public health resources, necessitating effective management strategies and ongoing surveillance to identify and control new cases [27]. Therefore, understanding the epidemiology and costs associated with these diseases is crucial for developing public health policies that improve care quality and optimize resource use for managing these severe and challenging conditions.

The healthcare system in Spain is paid for mostly by taxpayers through social security and provides comprehensive coverage to all residents and citizens, including free access to most medical services. Our study aimed to provide a comprehensive description and analysis of the demographic data, comorbidities, medical procedures, hospital outcomes, and admission costs of patients hospitalized with CJD and FFI in Spain from 2016 to 2022. By examining these factors, we sought to identify trends and differences in the disease burden and healthcare resource utilization associated with these two rare prion diseases. This analysis is intended to advance our understanding of the epidemiology and economic impact of CJD and FFI and to offer insights that can inform healthcare policies and improve management strategies for these conditions.

## 2. Materials and Methods

We performed a retrospective and observational epidemiological study using individual data from the Spanish National Hospital Discharge Database (SNHDD) for the period of 2016 to 2022. The SNHDD gathers patients’ information from all hospitals in the Spanish National Health Service. This comprises basic demographic data, up to 20 diagnoses and procedures, dates of admission and discharge, costs, and hospital outcomes. Coding was based on the International Classification of Diseases, Tenth Revision (ICD-10) [28].

As inclusion criteria, we selected all patients admitted with an ICD-10 code for CJD (A81.00, A81.01, and A81.09) and FFI (A81.83) in any diagnostic field. We excluded other associated prion diseases, such as Gerstmann–Sträussler–Scheinker syndrome (ICD-10 code A81.82), which is very rare and difficult to diagnose [29], and Kuru disease (ICD-10 code A81.81), owing to its endemic nature (limited to Papua New Guinea) [30].

We analyzed the total number of hospital admissions, whether a patient was hospitalized once or more, and the characteristics of individual patients.

The SNHDD is provided to us by the Spanish Ministry of Health with all personal identifiers deleted to warranty anonymity. Therefore, to identify those patients who were hospitalized more than once, we applied an algorithm including diagnosis of CJD or FFI, date of birth, sex, and place of residence (province). Therefore, if two or more patients hospitalized with an ICD10 code for CJD or FFI had exactly the same date of birth, and sex and lived in the same province, we would consider that these patients would be a single subject. The number of hospital admissions was categorized into one, two, and three or more admissions.

Age, sex, comorbidity, procedures carried out during the admission, hospital outcomes, and costs were the variables analyzed in our investigation.

Comorbidity was quantified using the Charlson Comorbidity Index (CCI) with the ICD-10 codes, as previously described [31]. We classified the study population into three categories: 0 (no illnesses), 1 (one illness), and 2 (two or more illnesses).

Furthermore, we examined other previously described long-term and potentially serious comorbidities of prion diseases, such as cancer [32], “mental, behavioral and neurodevelopmental disorders” [33], “extrapyramidal and movement disorders” [34], “Alzheimer’s disease and other degenerative diseases” [35], “encephalopathies and other nervous system disorders” [36], “hypertensive diseases”, “cerebrovascular diseases” [37], “pneumonia and other respiratory tract infections” [38], urinary tract infection [39], sepsis, and COVID-19 [40]. The ICD-10 codes for these diagnoses are shown in Table S1.

The procedures analyzed included computed tomography of the brain (ICD-10 code B020ZZZ), “measurement of central nervous electrical activity” (ICD10 code 4A00X4Z), “magnetic resonance imaging of brain” (ICD-10 code B030ZZZ), “drainage of the spinal canal” (ICD-10 code 009U3ZX), and “introduction of other anti-infective into peripheral vein, percutaneous approach” (ICD-10 code 3E03329).

Other hospital outcomes were in-hospital mortality (IHM), LOHS, and the costs of hospitalization per patient. The costs were compared by sex, age group, CCI, number of hospital admissions, and IHM.

### 2.1. Statistical Analysis

Quantitative data are expressed as the median with interquartile range (IQR) or the mean with standard deviation (SD). Qualitative data are expressed as frequencies and prevalence.

Statistical comparisons were performed using appropriate methods such as the χ^2^ test, Fisher’s exact test, the *t* test, or analysis of variance, as appropriate.

A multivariable logistic regression analysis was conducted to explore the association between IHM and the other study variables among CJD patients. This analysis was not possible with FFI patients owing to the very low number of cases.

Estimates were presented as odds ratios (ORs) with their 95% confidence intervals (CIs). All statistical analyses were performed using Stata version 28.1 (Stata, College Station, TX, USA). Statistical significance was set at *p* < 0.05 (two-tailed).

### 2.2. Ethical Aspects

The research protocol was submitted for approval to the Spanish Ministry of Health, which, after evaluation, provided us with the anonymized data.

As the SNHDD is a fully anonymized administrative database, the need for informed consent is waived according to Spanish law.

## 3. Results

Between 1 January 2016 and 31 December 2022, a total of 1063 hospital discharges of patients diagnosed with CJD or FFI were recorded in Spain.

Table 1 presents the results of hospitalizations with diagnoses of CJD and FFI in Spain from 2016 to 2022. A total of 1063 hospitalizations were identified, with 1020 for CJD and 43 for FFI. The annual distribution of hospitalizations for CJD remains relatively constant, while FFI shows a notable peak in 2017. Most patients with CJD are between 60 and 79 years old, whereas a large proportion of FFI patients are under 60 years old. LOHS is 13 days for CJD and 6 days for FFI. IHM is 36.37% for CJD and 32.56% for FFI. Of the 1020 hospitalizations with a diagnosis of CJD, there were 865 unique patients with this disease. The 43 admissions for FFI corresponded to 30 unique patients.

The characteristics of the 856 patients with CJD are shown in Table 2. As can be seen, 40.19% died in hospital.

The mean age of CJD patients was 68.38 years, with a higher mean age among those who died in hospital (70.58 years vs. 66.9 years; *p* < 0.001). Of the CJD patients, 107 (12.5%) had two admissions over the seven-year period and 25 (around 3%) had three or more admissions.

Among CJD patients, the most frequent coded comorbidities were “hypertensive diseases” (48.48%), “mental, behavioral and neurodevelopmental disorders” (45.79%), “episodic and paroxysmal disorders” (26.64%), and “pneumonia and other respiratory tract infections” (24.18%). The distribution of comorbidities with respect to IHM was statistically significant only for “encephalopathies and other nervous system disorders”, “pneumonia and other respiratory tract infections”, and sepsis. Patients with these comorbidities had a higher IHM than those without.

The median LOHS was 14 days, with a higher value among those who died (17.5 days vs. 12.5 days; *p* = 0.005).

Table 3 shows the characteristics of the 30 FFI patients hospitalized in Spain from 2016 to 2022 according to survival. Fourteen patients (46.67%) died during hospitalization. The mean age of the FFI patients was 53 years, and men accounted for 63.33%. No significant differences were found in the distribution of age or sex in relation to IHM.

Ten percent of FFI patients had two admissions and 6.67% had three or more.

The most frequent comorbidities in FFI patients were “mental, behavioral and neurodevelopmental disorders” (36.67%), “pneumonia and other respiratory tract infection” (30%), and “episodic and paroxysmal disorders” (16.67%). The overall LOHS was 7.5 days. None of the study variables analyzed were associated with IHM in FFI patients.

The multivariable logistic regression analysis to identify variables associated with IHM in patients diagnosed with CJD showed that, compared with the reference age group (<60 years), those aged 60–69 years (OR 1.43; 95%CI 0.92–2.23), 70–79 years (OR 2.16; 95%CI 1.37–3.41), and ≥80 years (OR 2.23; 95%CI 1.31–3.8) had a higher probability of IHM.

Comorbidities such as “pneumonia and other respiratory tract infections”, with an OR of 2.11 (95% CI: 1.47–3.01), and sepsis, with an OR of 5.59 (95% CI: 1.98–15.79), were also associated with IHM. Adjustment for potential confounding factors in the multivariable analysis revealed no significant change in IHM rates during the study period.

The most common procedures during a hospital admission with codes for CJD and FFI are shown in Table 4. “Drainage of spinal canal, percutaneous approach, diagnostic” was recorded in 12.59% of hospitalizations with CJD and in 6.12% of hospitalizations with CJD and FFI, followed by “Magnetic resonance imaging of brain” (9.68% and 6.12%) and “Measurement of central nervous electrical activity, external approach” (8.89% and 4.08%).

The estimated total cost of CJD and FFI patients over the seven-year study period was EUR 6,346,868, with EUR 6,135,609 (92.41%) caused by CJD and EUR 211,259 (7.59%) by FFI. Table 5 shows the cost per patient according to the study variables. The mean cost per patient was EUR 7167.77 for CJD and EUR 7041.97 for FFI.

Among CJD patients, costs were higher for those with a higher CCI value. CJD and FFI patients who were hospitalized more frequently had a higher mean cost than those hospitalized less frequently. Furthermore, costs for CJD and FFI patients who died during hospitalization were significantly higher than for those who survived.

## 4. Discussion

Our study aimed to provide a detailed epidemiological overview of prion diseases, specifically CJD and FFI, by analyzing hospital discharge data from the Spanish National Health System between 2016 and 2022. The ultimate goal was to elucidate the demographic characteristics, hospital outcomes, and economic impact of these diseases, as well as to explore the implications of our findings for future research and clinical practice.

From 1 January 2016 to 31 December 2022, there were 1063 hospital discharges of patients diagnosed with CJD (96%) and FFI (4%) in the Spanish National Health System. FFI patients were significantly younger than those with CJD (*p* < 0.001), with most CJD patients aged over 60 years and most FFI patients aged under 60. The LOHS was 13 days for CJD and six days for FFI. The common comorbidities detected in patients with CJD were “hypertensive diseases”, “Mental, Behavioral and Neurodevelopmental disorders”, and “Pneumonia and other respiratory tract infections”. Multivariable analysis confirmed that advanced age, pneumonia, and sepsis increased IHM significantly in CJD.

According to data from the website of the National Registry of Human Transmissible Spongiform Encephalopathies (NRHTSE) in Spain [41], the frequencies of reported cases between 2016 and 2021 were considerably lower than those of the cases of CJD and FFI identified in the SNHDD database. Specifically, 424 cases were reported to the NRHTSE between 2016 and 2021, whereas 856 cases were registered in the SNHDD from 2016 to 2022. These discrepancies may reflect a potential bias in case reporting to the NRHTSE or a bias in data coding in the SNHDD. For instance, a bias in the NRHTSE reporting could arise from the very stringent requirements for including a case in the registry, which might lead to underreporting of cases. Conversely, a bias in the SNHDD coding might occur if some cases are based only on unconfirmed clinical criteria due to stringent diagnostic criteria. The WHO has established rigorous diagnostic criteria for prion diseases, including the use of biomarkers such as the 14-3-3 protein and RT-QuIC assay [9]. While these tools have significantly advanced the diagnostic accuracy of sCJD by identifying neuronal damage and amplifying abnormal prion proteins [10,11], the RT-QuIC assay is not yet routinely applied in clinical practice in Spain. Consequently, the study period (2016–2022) may not capture long-term trends in prion disease epidemiology, and future application of these advanced diagnostic methods could uncover new biases in epidemiological data and significantly impact both the early detection and clinical management of prion diseases.

NRHTSE in Spain shows geographically uneven frequencies across different regions [18]. However, the overall incidence rates of prion diseases in Spain are comparable to those in other countries that have similar surveillance systems. Additionally, there are no notable differences in the clinical profiles of prion disease patients in Spain compared to those observed in other countries with well-established prion disease surveillance networks [42].

However, when the age and sex of CJD cases in the two previously mentioned Spanish registries are compared, the percentage of women is larger than that of men in both, and the age group with the highest prevalence of CJD is from 70 to 79 years.

To our knowledge, this retrospective epidemiological study is the first to address prevalent prion diseases (CJD and FFI) by examining hospital discharges of patients based on a database managed by the Spanish Ministry of Health. While the number of hospitalizations for both disorders did not change substantially during the years studied, there were differences in age for inpatient care, with FFI patients being younger. This is consistent with previous studies describing how FFI patients are younger than CJD patients [43].

In our study, an age range of hospital admission from 60 to 80 years was observed in CJD patients. However, in the literature, age data range from 60–70 years [44] to 50–70 years [45]. These discrepancies underscore the complexity of age-related trends in the manifestation of CJD, likely influenced by factors such as demographic differences, study methodologies, and temporal variations [46]. Further exploration into the underlying mechanisms driving these divergent patterns is necessary if we are to increase our understanding of the epidemiology of CJD and thus improve interventions and management strategies.

The present study identified a positive association between in-hospital survival and the presence of comorbid conditions related to the autonomic nervous system and metabolic encephalopathies, as well as other, unspecified conditions. The clinical complexity associated with these comorbid conditions could lead to heightened vigilance and medical care from health personnel, potentially facilitating the early detection of complications and more aggressive intervention. The relationship between CJD and comorbidities of the autonomic nervous system or unspecified encephalopathies is multifactorial and warrants further research to fully comprehend its clinical implications. As previously described, the comorbidities of pneumonia and sepsis are associated with mortality [47]. Sepsis is a critical condition in which the body reacts inappropriately to infection, which can occur in patients with CJD owing to immunological weakness associated with disease progression, significantly increasing the risk of mortality. Moreover, pneumonia is a frequent adverse event in patients with advanced neurological diseases [48,49], such as prion disease, because of dysphagia, being bedridden, and muscle weakness, all of which can lead to aspiration of gastric contents and bacterial colonization of the respiratory tract, thus increasing the likelihood of severe complications and death.

Our findings for CJD patients support the association between age and LOHS with IHM, suggesting that older age and longer LOHS are associated with increased IHM. However, this pattern does not appear to be consistent in patients diagnosed with FFI. These results suggest significant differences in mortality dynamics between the two prion diseases, emphasizing the need for clinical management approaches that are tailored to the disorder in question.

To our knowledge, neither LOHS nor IHM has previously been documented in FFI patients; therefore, it is not possible to make comparisons.

The variability in IHM data among CJD patients is notable. We reported that 40.19% died, while other authors have reported mortality rates ranging from 8.5% [45] to 53.5% [22]. The first study, involving a retrospective cohort, was based on the Healthcare Cost and Utilization Project (HCUP) database. Patients were selected using ICD-10 codes, with only diagnoses classified as definitive included. In the study by Bernardes et al., data recorded in a hospitalization database managed by the Health Services Authority of the Portuguese Ministry of Health were analyzed retrospectively, although the authors used ICD-9 codes. This considerable variation underscores the need for further research to identify the factors that may affect LOHS and IHM in CJD patients and for standardized approaches to assess outcomes across different clinical environments.

In our study population, “drainage of the spinal canal via a percutaneous approach” was the most frequent procedure. This was expected since the presence of proteins in cerebrospinal fluid (CSF) is a highly specific and sensitive biomarker for prion diseases [50]. Specifically, the presence of 14-3-3 proteins in CSF is included in the clinical criteria for CJD [51,52], and the real-time quaking-induced conversion (RT-QuIC) assay enhances the diagnostic accuracy of suspected prion diseases [9,53]. The other two most frequent procedures were “Measurement of central nervous electrical activity, external approach” and “Magnetic resonance imaging of brain”. Both were expected, since they are used in the diagnostic work-up for prion diseases. The diagnostic criteria for CJD of the World Health Organization include typical nervous electrical activity patterns [54,55] and magnetic resonance imaging findings, including basal ganglia hyperintensities [52], which have a diagnostic certainty level of probable [51].

The cost analysis in our study revealed a mean cost per admission with prion disease of around EUR 7200. Our findings differ significantly from those of previous studies. For instance, another study reported a much higher cost of USD 19,901 per patient admission [45], while Bakal et al. indicated an even higher cost per patient of USD 52,232, although their study considered annual costs instead of per-admission costs. These differences emphasize the variability in costs associated with the disease and highlight the importance of understanding methodological differences between studies when interpreting and comparing economic outcomes. Further investigation is warranted to elucidate the factors contributing to these discrepancies and thus provide a more comprehensive understanding of the economic burden of the condition.

Our research reveals limitations in the use of the SNHDD. The validity of coding prion diseases with ICD-10 has not been evaluated in the SNHDD. Indeed, achieving a successful early diagnosis of prion diseases can be a challenging task, especially in cases of rapid progression [56]. Unfortunately, the SNHDD does not collect detailed clinical information on the patients, such as diagnostic tests performed, symptom presentation, disease progression, or specific treatment approaches. These and other missing variables are potential confounding factors that could not be evaluated. In the future, the SNHDD must collect more specific data regarding the symptoms of prion diseases and specific biomarkers in order to reduce selection bias and improve the representativeness of the findings. Additionally, the consistency of the SNHDD depends on the quality of the discharge reports [57]. A further constraint of our study is the small sample size for FFI. It would be desirable to include a longer timeframe, which would allow for the analysis of temporal trends in the epidemiology and clinical features of these rare prion diseases. However, as the ICD-10 was implemented in the SNHDD in 2016 and the codes used by the previous ICD-9 are significantly different, we could not join the SNHDD databases prior to 2016 [28]. Future studies should analyze a longer time period to be more precise in detecting epidemiological variations patients, such as diagnostic tests performed, symptom presentation, disease progression, or specific treatment approaches. These and other missing variables are potential confounding factors that could not be evaluated. Regarding the analysis of IHM, we used logistic regression instead of survival analysis because we aimed to identify which variables present at the time of admission were associated with the probability of death during the hospital stay. The Cox model provides the probabilities of discharge alive (or death) as a function of time, whereas a logistic model gives the probability of the outcome at discharge as a dichotomous event, irrespective of time [58]. The average LOHS for patients in our study is short (median 13 days), which limits the Cox regression analysis’s ability to detect the effect of time on survival. The Cox regression for survival analysis would have been very appropriate if we had the variable for the date of disease diagnosis, which we unfortunately do not have.

One of the primary strengths of our study is the use of a comprehensive national database, which allowed us to gain a detailed understanding of the epidemiology and hospital outcomes of prion diseases across Spain [59]. This national coverage provides a robust and generalizable perspective on the prevalence, mortality, and associated costs of hospitalized CJD and FFI patients, which strengthens the validity of our findings and their applicability to a wide population. The algorithms used to identify patients with more than one admission are not precise, thus potentially overestimating the number of individual patients.

## 5. Conclusions

In conclusion, this epidemiological study shows the burden and hospital outcomes associated with prion diseases, specifically CJD and FFI, in Spanish hospitals from 2016 to 2022. Demographic distribution differed, with CJD mainly impacting individuals aged over 70 years, while FFI affects younger individuals. Both diseases have a high IHM, which is associated with age, pneumonia, and sepsis. The study also reveals high costs, thus highlighting the economic significance of these conditions. Nonetheless, limitations are recognized, and the differences between the cases reported by the NRHTSE and the SNHDD should be investigated.

Future investigations are needed, particularly regarding the incorporation of new biomarkers in diagnostic certainty criteria. This advancement could enhance the accuracy and reliability of diagnoses, thereby improving patient outcomes and facilitating more targeted treatments. Such research will be vital in advancing our understanding and management of prion diseases.

## Figures and Tables

**Table 1:** Hospital admission with a diagnosis code for Creutzfeldt–Jakob disease or fatal familial insomnia in Spain from 2016 to 2022 according to the study variables.

		CJD	FFI	Total
N		1020	43	1063
Years, *n* (%)	2016	123 (12.06)	3 (6.98)	126 (11.85)
	2017	160 (15.69)	13 (30.23)	173 (16.27)
	2018	132 (12.94)	7 (16.28)	139 (13.07)
	2019	150 (14.71)	7 (16.28)	157 (14.77)
	2020	155 (15.2)	2 (4.65)	157 (14.77)
	2021	157 (15.39)	6 (13.95)	163 (15.33)
	2022	143 (14.02)	5 (11.63)	148 (13.9)
Age, mean (sd)		67.93 (13.24)	48.93 (17.4)	58.43 (14.28)
Age groups in years, *n* (%)	<60	195 (19.12)	34 (79.07)	229 (21.54)
	60–69	321 (31.47)	2 (4.65)	323 (30.38)
	70–79	340 (33.33)	4 (9.3)	344 (32.61)
	≥80	164 (16.08)	3 (6.98)	167 (15.71)
Sex, *n* (%)	Men	492 (48.24)	29 (67.44)	521 (49.01)
	Women	528 (51.76)	14 (32.56)	542 (50.99)
LOHS, median (IQR)		13 (18)	6 (12)	9.5 (15)
In-hospital mortality, *n* (%)	Yes	371 (36.37)	14 (32.56)	385 (36.22)
	No	649 (63.63)	29 (67.44)	678 (63.78)

CJD: Creutzfeldt–Jakob disease. FFI: fatal familial insomnia. LOHS: length of hospital stay. N: number of hospital admissions. IQR: interquartile range.

**Table 2:** Distribution according to study variables of patients with a diagnosis of Creutzfeldt–Jakob disease in Spain from 2016 to 2022, according to survival to hospital admission.

		Total	Survived	Died	*p*-Value
Number of individuals, *n* (%)		856 (100)	512 (59.81)	344 (40.19)	
Age. mean (SD)		68.38 (13.09)	66.9 (14.55)	70.58 (10.17)	<0.001
Age group in years, *n* (%)	<60	157 (18.34)	105 (66.88)	52 (33.12)	0.043
	60–69	265 (30.96)	167 (63.02)	98 (36.98)	
	70–79	285 (33.29)	159 (55.79)	126 (44.21)	
	≥80	149 (17.41)	81 (54.36)	68 (45.64)	
Sex, *n* (%)	Men	406 (47.43)	236 (58.13)	170 (41.87)	0.339
	Women	450 (52.57)	276 (61.33)	174 (38.67)	
Number of hospital admissions, *n* (%)	1	724 (84.58)	425 (58.7)	299 (41.3)	0.036
	2	107 (12.5)	66 (61.68)	41 (38.32)	
	≥3	25 (2.92)	21 (84)	4 (16)	
CCI, *n* (%)	0	354 (41.36)	215 (60.73)	139 (39.27)	0.842
	1	289 (33.76)	172 (59.52)	117 (40.48)	
	≥2	213 (24.88)	125 (58.69)	88 (41.31)	
CCI, mean (SD)		1.05 (1.33)	1.03 (1.31)	1.09 (1.35)	0.482
Malignant neoplasms, *n* (%)	Yes	28 (3.27)	15 (53.57)	13 (46.43)	0.508
Mental, behavioral, and neurodevelopmental disorders, *n* (%)	Yes	392 (45.79)	238 (60.71)	154 (39.29)	0.600
Extrapyramidal and movement disorders, *n* (%)	Yes	100 (11.68)	66 (66)	34 (34)	0.211
Alzheimer’s disease and other degenerative diseases, *n* (%)	Yes	93 (10.86)	60 (64.52)	33 (35.48)	0.376
Episodic and paroxysmal disorders, *n* (%)	Yes	228 (26.64)	129 (56.58)	99 (43.42)	0.239
Encephalopathies and other nervous system disorders, *n* (%)	Yes	188 (21.96)	101 (53.72)	87 (46.28)	0.040 *
Hypertensive diseases, *n* (%)	Yes	415 (48.48)	249 (60)	166 (40)	0.984
Cerebrovascular diseases, *n* (%)	Yes	73 (8.53)	38 (52.05)	35 (47.95)	0.169
Pneumonia and other respiratory tract infections	Yes	207 (24.18)	97 (46.86)	110 (53.14)	<0.001
Urinary tract infection, *n* (%)	Yes	131 (15.3)	80 (61.07)	51 (38.93)	0.799
Sepsis, *n* (%)	Yes	25 (2.92)	5 (20)	20 (80)	<0.001
COVID-19 *, *n* (%)	Yes	28 (3.27)	16 (57.14)	12 (42.86)	0.787
LOHS, median (IQR)		14 (18)	12.5 (16)	17.5 (22)	0.005

* Data for COVID-19 are only for the years 2000 to 2022. CCI: Charlson Comorbidity Index. LOHS: length of hospital stay. IQR: interquartile range.

**Table 3:** Distribution according to the study variables of patients with a diagnosis of fatal familial insomnia in Spain from 2016 to 2022, according to survival to hospital admission.

		Total	Survived	Died	*p*-Value
Number of individuals, *n* (%)		30 (100)	16 (53.33)	14 (46.67)	
Age. mean (SD)		53 (18.91)	52 (21.16)	54.14 (16.7)	0.765
Age group in years, *n* (%)	<60	21 (70)	11 (52.38)	10 (47.62)	0.969
	60–69	2 (6.67)	1 (50)	1 (50)	
	70–79	4 (13.33)	2 (50)	2 (50)	
	≥80	3 (10)	2 (66.67)	1 (33.33)	
Sex, *n* (%)	Men	19 (63.33)	9 (47.37)	10 (52.63)	0.389
	Women	11 (36.67)	7 (63.64)	4 (36.36)	
Number of hospital admissions, *n* (%)	1	25 (83.33)	14 (56)	11 (44)	0.276
	2	3 (10)	2 (66.67)	1 (33.33)	
	≥3	2 (6.67)	0 (0)	2 (100)	
CCI, *n* (%)	0	16 (53.33)	7 (43.75)	9 (56.25)	0.146
	1	7 (23.33)	3 (42.86)	4 (57.14)	
	≥2	7 (23.33)	6 (85.71)	1 (14.29)	
CCI, mean (SD)		0.77 (0.97)	1.06 (1.12)	0.43 (0.65)	0.126
Malignant neoplasms, *n* (%)	Yes	0 (0)	0 (0)	0 (0)	NA
Mental, behavioral, and neurodevelopmental disorders, *n* (%)	Yes	11 (36.67)	9 (81.82)	2 (18.18)	0.097
Extrapyramidal and movement disorders, *n* (%)	Yes	3 (10)	1 (33.33)	2 (66.67)	0.287
Alzheimer’s disease and other degenerative diseases, *n* (%)	Yes	1 (3.33)	1 (100)	0 (0)	0.356
Episodic and paroxysmal disorders, *n* (%)	Yes	5 (16.67)	4 (80)	1 (20)	0.264
Encephalopathies and other nervous system disorders, *n* (%)	Yes	4 (13.33)	2 (50)	2 (50)	0.900
Hypertensive diseases, *n* (%)	Yes	6 (20)	2 (33.33)	4 (66.67)	0.371
Cerebrovascular diseases, *n* (%)	Yes	1 (3.33)	1 (100)	0 (0)	0.356
Pneumonia and other respiratory tract infections	Yes	9 (30)	5 (55.56)	4 (44.44)	0.907
Urinary tract infection, *n* (%)	Yes	1 (3.33)	0 (0)	1 (100)	0.294
Sepsis, *n* (%)	Yes	1 (3.33)	0 (0)	1 (100)	0.294
COVID-19 *, *n* (%)	Yes	0 (0)	0 (0)	0 (0)	NA
LOHS, median (IQR)		7 (9)	7.5 (16)	6 (6)	0.456

* Data for COVID-19 are only for the years 2000 to 2022. CCI: Charlson Comorbidity Index. LOHS: length of hospital stay. IQR: interquartile range. NA: not available.

**Table 4:** Most common procedures conducted during hospital admission with a diagnosis code for Creutzfeldt–Jakob disease or fatal familial insomnia in Spain from 2016 to 2022.

Procedures	CJD	FFI	Total Prions
Introduction of other anti-infectives into the peripheral vein, percutaneous approach, *n* (%)	129 (3.76)	4 (4.08)	133 (3.76)
Computerized tomography of the brain, *n* (%)	205 (5.98)	1 (1.02)	206 (5.84)
Measurement of central nervous electrical activity, external approach, *n* (%)	305 (8.89)	4 (4.08)	309 (8.75)
Magnetic resonance imaging of the brain, *n* (%)	332 (9.68)	6 (6.12)	338 (9.58)
Drainage of spinal canal, percutaneous approach, diagnostic, *n* (%)	432 (12.59)	6 (6.12)	438 (12.41)
Total, procedures, *n*	3430	98	3528

CJD: Creutzfeldt–Jakob disease. FFI: fatal familial insomnia.

**Table 5:** Hospitalization cost per patient with a diagnosis code for Creutzfeldt–Jakob disease or fatal familial insomnia in Spain from 2016 to 2022.

		CJD	FFI
		Euros	Euros
Total, mean (sd)		7167.77 (6793.57)	7041.97 (6444.29)
Age group, mean (sd)	<60 years	8365.73 (11266.53)	7421.1 (7270.74)
	60–69 years	6677.72 (4477.92)	3685.47 (1637.65)
	70–79 years	7143.04 (5515.63)	5635.61 (2775.03)
	≥80 years	6824.36 (6050.68)	8500.86 (6380.93)
Sex, mean (sd)	Men	7584.05 (7828.77)	6844.27 (7450.29)
	Women	6792.19 (5683.63)	7383.45 (4507.64)
Charlson Comorbidity Index, mean (sd)	0	6362.32 (5539.71)	7455.81 (8328.39)
	1	7194.86 (6568.88)	5785.12 (2116.48)
	≥2	8469.64 (8580.74)	7352.9 (4519.49)
Number of hospital admissions, mean (sd)	1	6363.24 (6637.2)	5278.57 (2789.91)
	2	10,235.48 (2872.74)	9404.68 (756.92)
	≥3	17,337.22 (10,556.95)	25,540.43 (15,411.41)
In-hospital mortality, mean (sd)	Yes	8083.43 (8312.81)	8272.56 (8627.1)
	No	6552.56 (5467.57)	5965.2 (3624.33)

CJD: Creutzfeldt–Jakob disease. FFI: fatal familial insomnia. Sd: standard deviation.

## Data Availability

According to the contract signed with the Spanish Ministry of Health and Social Services, which provided access to the databases from the Spanish National Hospital Database, we cannot share the databases with any other investigator, and we have to destroy the databases once the investigation has concluded. Consequently, we cannot upload the databases to any public repository. However, any investigator can apply for access to the databases by filling out the questionnaire available at https://www.sanidad.gob.es/estadEstudios/estadisticas/estadisticas/estMinisterio/SolicitudCMBD.htm (accessed on 20 June 2024). All other relevant data are included in the paper.

## Supplementary Materials

The following supporting information can be downloaded at: https://www.mdpi.com/article/10.3390/jcm13154401/s1. Table S1: International Classification of Diseases, Tenth Revision diagnosis codes used in this investigation.
