# Supplementary material for: Creutzfeldt–Jakob Disease and Fatal Familial Insomnia: Demographics and In-Hospital Mortality in Spain
Source: J Clin Med. 2024 Jul 27;13(15):4401. doi: 10.3390/jcm13154401 (PMC11312717; doi:10.3390/jcm13154401)
Supplement: Supplementary file 1 [file jcm-13-04401-s001.zip › jcm-3111313-supplementary.pdf]

Supplementary Materials

Table S1. International Classification of Diseases, Tenth Revision diagnosis codes used in this investigation.

DIAGNOSIS	IC10 CODES
Creutzfeldt-Jakob disease	A81.00, A81.9, A81.09
Fatal familial insomnia	A81.83
Malignant neoplams	C00-C96
Diabetes	E11.00, E11.10, E11.52, E11.621, E11.649, E11.65
Mental and behavioral disorders	F00-F99
Extrapyramidal and movement disorders	G20-G26, R26.0 y R26.9
Alzheimer's disease and other degenerative diseases	G30-G31
Episodic and paroxysmal disorders	G40-G47
Encephalopathies and other nervous system disorders	G90-G99
Hipertensive diseases	I10-I16
Cerebrovascular diseases	I60-I69
Pneumonia and other respiratory tract infections	J00-J22
Urinary tract infection	N39.0
Sepsis	A41
COVID-19	U07.1
